# Physical activity and daily steps cut offs points for overweight/obesity prevention among eight Latin American countries

**DOI:** 10.1038/s41598-022-23586-y

**Published:** 2022-11-05

**Authors:** Paloma Ferrero-Hernández, Claudio Farías-Valenzuela, Emilio Jofré-Saldía, Adilson Marques, Irina Kovalskys, Georgina Gómez, Attilio Rigotti, Lilia Yadira Cortés, Martha Yépez García, Rossina G. Pareja, Marianella Herrera-Cuenca, Mauro Fisberg, Danilo R. Silva, Kabir P. Sadarangani, Gerson Ferrari

**Affiliations:** 1 Facultad de Educación y Cultura, Universidad SEK, Santiago, Chile; 2 Instituto del Deporte, Universidad de las Américas, Santiago, Chile; 3 Facultad de Educación y Ciencias Sociales, Instituto del Deporte y Bienestar, Universidad Andres Bello, 7550000 Santiago, Chile; 4 Instituto de Ciencias de la Salud, Universidad de O’Higgins, Rancagua, Chile; 5 CIPER, Faculdade de Motricidade Humana, Universidade de Lisboa, Lisbon, Portugal; 6 ISAMB, Faculdade de Medicina, Universidade de Lisboa, Lisbon, Portugal; 7 Carrera de Nutrición, Facultad de Ciencias Médicas, Pontificia Universidad Católica Argentina, Buenos Aires, Argentina; 8 Departamento de Bioquímica, Escuela de Medicina, Universidad de Costa Rica, San José, Costa Rica; 9 Centro de Nutrición Molecular y Enfermedades Crónicas, Departamento de Nutrición, Diabetes y Metabolismo, Escuela de Medicina, Pontificia Universidad Católica, Santiago, Chile; 10 Departamento de Nutrición y Bioquímica, Pontificia Universidad Javeriana, Bogotá, Colombia; 11 Colégio de Ciencias de la Salud, Universidad San Francisco de Quito, Quito, Ecuador; 12 Instituto de Investigación Nutricional, La Molina, Lima Peru; 13 Centro de Estudios del Desarrollo, Universidad Central de Venezuela (CENDES-UCV)/Fundación Bengoa, Caracas, Venezuela; 14 Centro de Excelência em Nutrição e Dificuldades Alimentares (CENDA), Instituto Pensi, Fundação José Luiz Egydio Setubal, Hospital Infantil Sabará, São Paulo, Brazil; 15 Departamento de Pediatria da Universidade Federal de São Paulo, São Paulo, Brazil; 16 Department of Physical Education, Federal University of Sergipe − UFS, São Cristóvão, Brazil; 17 School of Kinesiology, Faculty of Health and Dentistry, Universidad Diego Portales, 8370057 Santiago, Chile; 18 Escuela de Kinesiología, Facultad de Odontología y Ciencias de la Rehabilitación, Universidad San Sebastián, Providencia, Santiago, Chile; 19 Escuela de Ciencias de la Actividad Física, el Deporte y la Salud, Universidad de Santiago de Chile (USACH), Santiago, Chile; 20 Faculty of Health Sciences, Universidad Autónoma de Chile, Av. Pedro de Valdivia 425, Providencia, 7500912 Santiago, Chile; 21 Department of Sports and Computer Science, Universidad Pablo de Olavide (UPO), 41013 Seville, Spain

**Keywords:** Metabolic disorders, Nutrition disorders

## Abstract

This study aims to establish cut-off points for the number of minutes of physical activity intensity and the number of daily steps that identify overweight/obesity in adolescents, adults, and older adults. This study examined data from 2737 participants. Physical activity intensity and the number of daily steps were assessed using GT3X+ ActiGraph model accelerometers. Body mass index, waist-to-height ratio, and waist-to-hip ratio were used as indicators of overweight/obesity. The cut-off points for moderate-to-vigorous physical activity for the prevention of overweight/obesity according to body mass index in women ranged from 15.1 to 30.2 min/day; in men, the values were from 15.4 to 33.8 min/day. The lowest cut-off point for daily steps was established in the adolescent group for women and men (7304 and 5162). The highest value in women was 11,412 (51–65 years) and 13,234 in men (18–30 years). Results from measurements different from BMI, show average cut-off points for moderate-to-vigorous physical activity and daily steps of 29.1/8348 and 43.5/10,456 according to waist-to-height ratio; and results of 29.3/11,900 and 44.3/11,056 according to the waist-to-hip ratio; in women and men respectively. A more specific recommendation of physical activity and daily steps adjusted by sex and age range is suggested to prevent overweight/obesity.

## Introduction

Obesity is a multifactorial disease that has grown in recent decades, with almost a third of the global population been classified as overweight/obese^1^. In Latin America, the prevalence of people with overweight/obesity is higher than in the rest of the world^2^, where two-thirds of women and half of men are overweight/obese in some countries, such as Chile and Mexico^3^. Overweight/obesity have been associated with metabolic diseases, increasing the risk of morbidity and mortality in the population with this condition^4, 5^.

Most epidemiological studies on obesity are based on the body mass index (BMI), which is generally accepted as a strong predictor of mortality^6, 7^ and although this conventional measurement of obesity has some benefits, there is concern that not all individuals at risk of obesity-associated medical conditions are being identified. Also, the whole-body fat percentage and specifically visceral adipose tissue mass are correlated and potentially implicated in disease development, but are not fully accounted for through BMI evaluation^8^. For this reason, in addition to BMI, other measurements of visceral adiposity, such as waist-to-height ratio (WHtR) and waist-hip ratio (WHR), have been validated as predictors of cardiovascular risk and mortality^9^. Therefore, health professionals should consider these measurements^10^. Evidence suggests that the WHtR is a stronger diagnostic indicator of overweight/obesity than the BMI or the WHR^11^; however, the WHR has also shown a significant association with the prevalence of hypertension and type 2 diabetes^12^.

Among the determinants of overweight/obesity, moderate-to-vigorous physical activity (MVPA) has been inversely associated with weight gain^13^. In addition to the minutes/intensity approach, the number of steps per day has also been used as indicator and target to achieve the benefits of physical activity (PA), being widely recommended at least 10,000 steps per day to maintain good health in adults^14^. However, less clear evidence is available to support the step-based recommendation. In adolescents, a threshold of 11,111 daily steps have been suggested as a step-based recommendation related to both PA and sedentary behavior thresholds, demonstrating a healthier cardiorespiratory fitness profile compared to their sedentary peers^15^. In this context, although a greater number of daily steps (8000–12,000) has been associated with lower mortality from all causes, a recent meta-analysis of 15 international cohorts studies suggested a plateau in the mortality risk reduction from 6000–8000 steps per day for older adults (≥ 60 years) and from 8000 to 10,000 steps per day for younger adults^16^. However, these recommendations are not specific for weigh management and do not consider samples from different sexes and age-groups out of the developed countries, which can influence the results analysis considering differences by age in functional capacity needs and PA recommendations. Thus, the aim of this study is to establish cut-off points for the number of minutes of MPA, VPA, MVPA, and the number of steps that identify overweight/obesity in adolescents, adults, and older adults from eight Latin American countries.

## Methods

### Study design and sample

This multinational cross-sectional study was obtained from the Latin American Study of Nutrition and Health (ELANS), which evaluated aspects of nutrition, PA, and sociodemographic characteristics in eight Latin American countries (Argentina, Brazil, Chile, Colombia, Costa Rica, Ecuador, Peru, and Venezuela). The survey was conducted from 2014 to 2015 using a complex, multistage, multisampling design, stratified by clusters. A random selection was made according to the probability proportional to size method. The survey included only participants from urban areas. Full details of ELANS can be found at https://www.elansstudy.com and in other previously published studies^17^.

In total, 92 cities participated in the ELANS study (from seven to 23 cities in each country). Respondents were selected from primary sampling unit areas (e.g., counties, townships, neighborhoods, suburbs, etc.). For the selection of households, a systematic 4-step randomization was implemented by establishing an interval selection: (1) the total urban population was used to proportionally describe the main regions and select cities that represent each region, (2) the samplers points (survey tranches) from each city were randomly designated, (3) groups of households were selected from each sampling unit, and (4) the designated respondent within each household was selected using the birthday method. A stratified recruitment participants was carried out in each country according to gender, age, and socioeconomic status (SES). The ELANS design and sample size have been described elsewhere^18^.

A total of 10,134 people (15.0–65.0 years of age) were invited to participate in the ELANS study. However, 9218 (4809 women) were included in the participants. In this study, participants who used accelerometers were considered according to gender, age, and SES ranges, thus ensuring a representative subsample^18^. A total of 2737 people (29.6% of the total sample) was considered in the current study, corresponding to all participants who had complete information from accelerometers regarding PA and daily steps and also had all the data on obesity indicators^19^. Participants with incomplete PA or anthropometric measurements data were excluded from the database.

All participants had to provide written informed consent before participating in the study, who voluntarily agreed to participate in the study and gave their permission for the future use of the recorded data. The ELANS protocol complies with the guidelines enunciated in the Declaration of Helsinki (2014) and has the approval of the ethics committee of the Western Institutional Review Board (#20140605), and registered with Clinical Trials (#NCT02226627). Also, a regional ethics committee approved the study in each country.

### Overweight/obesity indicators

The indicators of overweight/obesity used in the present study were body weight (kg), height (cm), waist circumference (cm), and hip circumference (HC; cm), which were evaluated according to standardized protocols^17, 20^. Each measurement was evaluated twice for greater precision, and an average of both was used for the analysis. Body weight and height were measured with participants wearing light clothing and without shoes, using an electronic scale and a portable stadiometer, respectively. Circumferences were measured with an inelastic tape to the nearest 0.1 cm. The midpoint between the last rib and the iliac crest was considered to measure the waist circumference. HC was measured at the largest protuberance level at the buttocks level, without pressing the soft tissues.

The BMI (weight (kg)/height (m^2^)), WHtR (waist circumference (cm)/height (cm)), and WHR (waist circumference (cm)/HC (cm)) indices were calculated in relation to the absolute values. The categorical BMI of adolescent participants was derived from the World Health Organization reference curves for age and sex^21^. Adults with a BMI ≥ 25.0 were classified as overweight/obese^22^. For WHtR and WHR, the cut-off points were ≥ 0.55 for adolescents and adults of both sexes; ≥ 1.0 for men and ≥ 0.85 for women^23, 24^. Participants were classified as eutrophic and overweight/obese.

### Accelerometry

GT3X+ ActiGraph model accelerometers (Pensacola, FL, USA) were used to assess PA intensity (MPA, VPA, and MVPA) and daily steps. Previous studies have widely documented its reliability and validity^25, 26^.

The accelerometers were given to the participants on a first visit, including a daily report for the following seven days, in which participants were instructed to complete a daily log to report the time they put the accelerometer belt on and the time when it was removed. The information they took in their daily logs, contributed to identify potential problems that could emerge from accelerometers use. They were removed on a second home visit. Participants were asked to wear the device on an elastic belt at hip level in the midaxillary line when they were awake and remove it when sleeping, showering, or swimming only, with specific instructions of removing the device only when going to sleep and wearing it immediately after waking up, without specifying the exact time. Excluding overnight sleep time, wake time without use was defined as any sequence of at least 60 consecutive minutes with zero activity. Data were collected at a sampling rate of 30 Hz and downloaded in 60-s time periods, then analyzed using ActiLife software (V6.0; ActiGraph, Pensacola, FL)^27^.

The data included and analyzed in the study corresponded to those with at least 10 h of recording for five days, including at least one weekend day^28^. Cut-off points considered for this study were taken from adult population for standardization purposes, considering that there was not pediatric population who participated from the study. Cut-off points were stablished at: 1952–5724 activity counts/min as MPA, 5725–9498 activity counts/min as VPA, and ≥ 1952 activity counts/min as MVPA (Troiano et al., 2008). In addition, we evaluated the number of daily steps. The mean absolute percent error of the GT3X+ accelerometer to measure daily steps is 14%^29^.

### Sociodemographic variables

The selection of respondents within a household was made at each country's level, considering the variables of interest. The participants of this study were stratified by sex, country, age group and SES. Age was categorized into the following groups: 15–17, 18–30, 31–50 and 51–65 years according to data collected from the ELANS study and group similarities regarding characteristics and PA recommendations. Regarding SES, this was evaluated by questionnaire using a country-dependent format and based on the legislative requirements or established local standard layouts. Three classification levels (low, medium, and high) were considered for all countries^30^, comparing the equivalized per-person income of each country/household with established thresholds for Latin Americans, drawn from national indexes used in each country.

### Statistical analysis

The sample's weighting was applied at each country's level considering gender, age, and SES^31^. All calculations were performed using SPSS version 26 software (SPSS Inc., IBM Corp., Armonk, New York, NY, USA). The Kolmogorov–Smirnov test was performed to evaluate the distribution of the data. For continuous variables, mean and standard deviation (SD) were presented; and categorical variables were expressed in frequency and percentage. The t-Student test for independent samples and Chi-square were used to compare the variables between the sexes. For non-parametric data (MPA, VPA, MVPA, and daily steps), the median and the 25th and 75th percentiles were also presented.

The predictive power and cut-off points of MPA, VPA, MVPA (min/day), and daily steps to prevent overweight/obesity were identified using Receiver Operating Characteristic (ROC) curves. We identified the total area under the ROC curve between the number of MPA, VPA, MVPA (min/day), daily steps and the prevention of overweight/obesity for BMI, WHtR and WHR. The greater the area under the ROC curve, the greater the discriminatory power, and a 95% confidence interval (95% CI) was also used. The 95% CI calculation determines whether the predictive capacity is not due to chance, and its limit must be greater than 0.50^32^. We then calculated sensitivity and specificity and cut-off points for MPA, VPA, MVPA, and the number of daily steps for prevention of overweight/obesity. Participants with low weight according to BMI were excluded from the ROC curve. Due to the significant difference in PA between the sexes, the results were presented for men and women separately. The significance of *p* < 0.05 was considered.

### Ethics approval

Ethical approval was approved by the Western Institutional Review Board (#20140605), and by regional ethical review boards of the participating institutions belonging to each country. The ELANS protocol is registered at Clinical Trials #NCT02226627.

### Consent to participate

Written informed consent/assent was obtained from all individuals before commencing the study.

## Results

Overall, 2737 people (52.2% women) with a mean age of 36.4 years (SD: 14.1) participated. Significant differences (*p* < 0.05) were found between the sexes for age, anthropometric measurements (body weight, height, BMI, waist circumference, HC, WHtR, and WHR), PA, and daily steps. On the other hand, no differences were found between the sexes only for SES and BMI categories (Table 1).

**Table 1:** Descriptive analysis for sociodemographic variables, anthropometric measurements and physical activity according to sex.

Variable	Total (n = 2737)	Women (n = 1430)	Men (n = 1307)	*p*
**Age (years)—Mean (SD)**	36.4 (14.1)	37.8 (14.0)	35.1 (14.2)	< 0.001^a^
**Age categories (years)—n (%)**				< 0.001^b^
15–17	213 (7.8)	89 (6.2)	124 (9.5)	
18–30	855 (31.2)	412 (28.9)	443 (33.9)	
31–50	1105 (40.4)	594 (41.5)	511 (39.1)	
51–65	564 (20.6)	335 (23.4)	229 (17.5)	
**Socioeconomic status—n (%)**				0.670^b^
Low	1401 (51.2)	739 (51.7)	662 (50.7)	
Medium	1062 (38.8)	544 (38.0)	518 (39.6)	
High	274 (10.0)	147 (10.3)	127 (9.7)	
**Anthropometry—Mean (SD)**				
Body weight (kg)	71.5 (15.9)	67.6 (14.6)	75.8 (16.1)	< 0.001^a^
Height (cm)	163.0 (9.5)	156.8 (6.8)	169.8 (7.2)	< 0.001^a^
BMI (kg/m^2^)	26.9 (5.4)	27.5 (5.7)	26.2 (5.1)	< 0.001^a^
Waist circumference (cm)	88.4 (14.1)	87.4 (13.8)	89.5 (14.3)	< 0.001^a^
Hip circumference (cm)	100.3 (11.3)	102.0 (11.6)	98.5 (10.7)	< 0.001^a^
WHtR	0.54 (0.09)	0.55 (0.09)	0.52 (0.08)	< 0.001^a^
WHR	0.88 (0.83)	0.85 (0.08)	0.90 (0.07)	< 0.001^a^
**BMI categories—n (%)**				0.514^b^
Underweight	72 (2.6)	36 (2.5)	36 (2.8)	
Eutrophic	993 (36.3)	470 (32.9)	523 (40)	
Overweight/obesity	1672 (61.1)	924 (64.6)	748 (57.2)	
**WHtR categories—n (%)**				< 0.001^b^
Eutrophic	1489 (54.4)	676 (47.3)	813 (62.2)	
Overweight/obesity	1247 (45.6)	753 (52.7)	494 (37.8)	
**WHR categories—n (%)**				< 0.001^b^
Eutrophic	1829 (66.8)	662 (46.3)	1167 (89.3)	
Overweight/obesity	908 (33.2)	768 (53.7)	140 (10.7)	
**MPA (min/day)**				
Mean (SD)	34.1 (24.5)	27.9 (19.2)	40.9 (27.7)	< 0.001^a^
Median (P25-P75)	28.2 (16.4–46.1)	23.5 (14.0–38.1)	35.2 (20.6–56.1)	
**VPA (min/day)**				
Mean (SD)	0.68 (2.2)	0.30 (1.4)	1.07 (2.8)	< 0.001^a^
Median (P25-P75)	0.00 (0.00–0.28)	0.00 (0.00–0.14)	0.14 (0.00–0.83)	
**MVPA (min/day)**				
Mean (SD)	34.8 (25.3)	28.2 (19.5)	42.0 (28.8)	< 0.001^a^
Median (P25-P75)	28.8 (16.5–47.1)	23.6 (14.1–38.4)	36.1 (21.0–57.2)	
**Daily steps**				
Mean (SD)	10,654.2 (5155.4)	10,076.8 (4774.2)	11,285.1 (5474.6)	< 0.001^a^
Median (P25-P75)	9636.7 (6689.8–13,922.8)	9095.0 (6340.1–13,063.8)	10,233.8 (7029.1–14,564.6)	

BMI, body mass index; WHtR, waist-to-height ratio; WHR, waist-hip ratio; MPA, moderate physical activity; VPA, vigorous physical activity; MVPA, moderate-to-vigorous physical activity.

^a^Significance value of t-student for independent samples.

^b^Chi-square test significance value.

Comparing by sex and age groups, women showed significantly higher BMI values than men (31–50 and 51–65 years). Furthermore, significant differences were found between the sexes for WHtR, WHR, MPA, and MVPA in all age categories. On average, men took more steps per day (*p* < 0.05) than women in the 18–30 and 31–50 groups (Table 2).

**Table 2:** Comparison (mean [95%CI] and SD) of overweight/obesity indicators and physical activity in relation to sex and age category.

Women								Men						
Age (years)	BMI (kg/m^2^)	WHtR	WHR	MPA (min/ day)	VPA (min/ day)	MVPA (min/ day)	Daily steps	BMI (kg/m^2^)	WHtR	WHR	MPA (min/ day)	VPA (min/ day)	MVPA (min/ day)	Daily steps
15–17	22.5 (3.5)	0.47* (0.06)	0.79* (0.06)	31.3* (21.8)	0.45 (1.1)	29.5* (20.6)	8999.6 (4646.1)	21.8 (3.5)	0.44 (0.05)	0.82 (0.05)	50.5 (31.5)	1.7 (3.0)	48.5 (29.7)	10,915.2 (5472.4)
18–30	25.3 (5.2)	0.51* (0.08)	0.82* (0.07)	29.2* (19.3)	0.4* (1.9)	27.3* (16.8)	9735.8* (4617.0)	25.0 (4.9)	0.49 (0.07)	0.87 (0.06)	45.2 (27.1)	1.6 (3.5)	44.4 (26.8)	11,396.2 (5304.7)
31–50	29.0* (5.7)	0.58* (0.08)	0.86* (0.07)	32.0* (22.3)	0.3 (1.9)	29.8* (21.0)	10,172.6* (4643.3)	27.6 (5.1)	0.55 (0.08)	0.93 (0.06)	41.4 (31.2)	0.9 (3.1)	40.2 (29.0)	11,329.1 (5542.4)
51–65	28.9* (5.0)	0.59* (0.08)	0.88* (0.07)	28.2* (21.8)	0.1 (0.7)	26.3* (19.6)	10,614.3 (5154.7)	27.8 (4.5)	0.57 (0.07)	0.96 (0.06)	39.8 (32.1)	0.4 (1.8)	38.0 (30.6)	11,172.3 (5669.6)

BMI, body mass index; WHtR, waist-to-height ratio; WHR, waist-hip ratio; MVPA, moderate-vigorous physical activity.

*Difference between men and women made with the t-Student test.

Tables 3, 4, and 5 show the results of the ROC curve analysis, establishing the cut-off points for MPA, VPA, MVPA (min/day), and daily steps in relation to BMI, WHtR, and WHR, according to sex and age. The area under the curve indicates the best values for each category according to the age range.

**Table 3:** Physical activity cut-off points (min/day) and number of steps according to sex and age categories in relation to BMI.

**Women**	**Age (years)**	**AUC**	**CI 95%**	**Sensibility (%)**	**Specificity (%)**	**MPA cut-off point (min/day)**
	15–17	.782	.639–.926	50.0	15.3	14.8
	18–30	.501	.421–.580	50.0	48.3	23.6
	31–50	.573	.514–.632	62.3	50.0	27.9
	51–65	.500	.411–.589	70.1	63.2	30.2
	**Age (years)**	**AUC**	**CI 95%**	**Sensibility (%)**	**Specificity (%)**	**VPA cut-off point (min/day)**
	15–17	.675	.501–.850	60.0	40.0	NA
	18–30	.548	.470–.627	85.9	75.6	0.24
	31–50	.531	.469–.592	84.6	78.9	0.21
	51–65	.511	.425–.597	92.1	92.6	0.15
	**Age (years)**	**AUC**	**CI 95%**	**Sensibility (%)**	**Specificity (%)**	**MVPA cut-off point (min/day)**
	15–17	.795	.204–.642	50.0	20.3	16.5
	18–30	.506	.508–.673	50.0	47.4	23.5
	31–50	.575	.579–.704	31.1	20.4	15.1
	51–65	.499	.529–.736	70.1	63.2	30.2
	**Age (years)**	**AUC**	**CI 95%**	**Sensibility (%)**	**Specificity (%)**	**Daily steps cut-off point**
	15–17	.720	.561–.879	83.3	40.7	7304
	18–30	.517	.435–.599	64.1	60.3	10,156
	31–50	.540	.527–.643	60.1	45.1	9544
	51–65	.573	.427–.603	70.1	60.3	11,412
**Men**	**Age (years)**	**AUC**	**CI 95%**	**Sensibility (%)**	**Specificity (%)**	**MPA cut-off point (min/day)**
	15–17	.424	.201–.646	25.0	11.0	14.0
	18–30	.588	.505–.670	50.8	39.1	33.0
	31–50	.639	.577–.701	76.2	50.3	40.7
	51–65	.632	.528–.736	70.4	60.3	40.0
	**Age (years)**	**AUC**	**CI 95%**	**Sensibility (%)**	**Specificity (%)**	**VPA cut-off point (min/day)**
	15–17	.512	.297–.728	50.0	41.5	0.38
	18–30	.608	.526–.690	72.9	50.6	0.53
	31–50	.588	.524–.652	92.5	80.0	1.0
	51–65	.500	.393–.608	79.6	77.6	0.24
	**Age (years)**	**AUC**	**CI 95%**	**Sensibility (%)**	**Specificity (%)**	**MVPA cut-off point (min/day)**
	15–17	.423	.661–.930	25.0	11.0	15.4
	18–30	.591	.427–.585	32.2	20.2	23.0
	31–50	.642	.516–.634	66.0	40.6	33.8
	51–65	.633	.411–.588	50.0	20.7	21.4
	**Age (years)**	**AUC**	**CI 95%**	**Sensibility (%)**	**Specificity (%)**	**Daily steps cut-off point**
	15–17	.454	.205–.704	25.0	11.0	5162
	18–30	.517	.431–.603	71.2	63.1	13,234
	31–50	.585	.475–.605	56.5	50.3	10,295
	51–65	.515	.467–.680	75.9	60.3	12,509

AUC, area under the curve; CI 95%, confidence interval, MPA, moderate physical activity, VPA, vigorous physical activity, MVPA, moderate vigorous physical activity.

**Table 4:** Physical activity cut-off points (min/day) and number of steps according to sex and age categories in relation to WHtR.

**Women**	**Age (years)**	**AUC**	**CI 95%**	**Sensibility (%)**	**Specificity (%)**	**MPA cut-off point (min/day)**
	15–17	.620	.412–.829	25.0	18.15	13.5
	18–30	.502	.443–.561	51.6	52.8	24.4
	31–50	.538	.490–.586	60.2	51.6	28.3
	51–65	.530	.457–.602	70.0	60.5	31.3
	**Age (years)**	**AUC**	**CI 95%**	**Sensibility (%)**	**Specificity (%)**	**VPA cut-off point (min/day)**
	15–17	.606	.404–.809	87.5	65.4	0.15
	18–30	.538	.479–.598	82.5	75.9	0.18
	31–50	.535	.486–.583	82.8	75.6	0.15
	51–65	.518	.446–.590	88.3	84.9	0.07
	**Age (years)**	**AUC**	**CI 95%**	**Sensibility (%)**	**Specificity (%)**	**MVPA cut-off point (min/day)**
	15–17	.623	.410–.835	75.0	60.5	31.6
	18–30	.508	.449–.567	52.4	51.7	24.5
	31–50	.541	.493–.589	60.2	51.6	29.0
	51–65	.530	.457–.603	70.0	60.5	31.3
	**Age (years)**	**AUC**	**CI 95%**	**Sensibility (%)**	**Specificity (%)**	**Daily steps cut-off point**
	15–17	.613	.422–.803	37.5	35.8	6143
	18–30	.474	.413–.535	29.4	33.2	6876
	31–50	.566	.519–.613	66.1	60.2	11,247
	51–65	.586	.516–.657	50.2	34.9	9127
**Men**	**Age (years)**	**AUC**	**CI 95%**	**Sensibility (%)**	**Specificity (%)**	**MPA cut-off point (min/day)**
	15–17	.403	.174–.632	25.0	64.7	50.4
	18–30	.590	.512–.649	59.6	51.7	40.2
	31–50	.576	.527–.626	65.2	52.5	39.1
	51–65	.536	.460–.612	59.9	57.3	37.4
	**Age (years)**	**AUC**	**CI 95%**	**Sensibility (%)**	**Specificity (%)**	**VPA cut-off point (min/day)**
	15–17	.489	.286–.691	50.0	60.3	0.95
	18–30	.643	.580–.706	57.3	37.0	0.15
	31–50	.583	.533–.632	68.0	54.4	0.15
	51–65	.532	.453–.612	84.4	72.0	0.15
	**Age (years)**	**AUC**	**CI 95%**	**Sensibility (%)**	**Specificity (%)**	**MVPA cut-off point (min/day)**
	15–17	.405	.179–.631	25.0	62.1	50.9
	18–30	.589	.521–.657	70.8	55.9	46.7
	31–50	.581	.531–.630	64.8	51.0	39.0
	51–65	.538	.461–.614	59.9	57.3	37.5
	**Age (years)**	**AUC**	**CI 95%**	**Sensibility (%)**	**Specificity (%)**	**Daily steps cut-off point**
	15–17	.456	.219–.692	25.0	39.7	8830
	18–30	.542	.475–.610	50.6	48.0	10,249
	31–50	.552	.502–.602	70.0	63.6	13,015
	51–65	.552	.475–.603	50.3	45.1	9732

AUC, area under the curve; CI 95%, confidence interval; MPA, moderate physical activity; VPA, vigorous physical activity; MVPA, moderate-to-vigorous physical activity.

**Table 5:** Physical activity cut-off points (min/day) and number of steps according to sex and age categories in relation to WHR.

**Women**	**Age (years)**	**AUC**	**CI 95%**	**Sensibility (%)**	**Specificity (%)**	**MPA cut-off point (min/day)**
	15–17	.586	.437–.699	52.4	48.5	22.1
	18–30	.471	.414–.528	80.3	84.5	42.9
	31–50	.547	.499–.594	50.4	40.6	23.4
	51–65	.540	.472–.608	49.4	49.5	21.3
	**Age (years)**	**AUC**	**CI 95%**	**Sensibility (%)**	**Specificity (%)**	**VPA cut-off point (min/day)**
	15–17	.572	.439–.706	61.9	52.9	0.0
	18–30	.524	.466–.581	91.2	89.4	0.5
	31–50	.529	.481–.577	75.5	70.5	0.0
	51–65	.519	.449–.589	88.4	84.9	0.0
	**Age (years)**	**AUC**	**CI 95%**	**Sensibility (%)**	**Specificity (%)**	**MVPA cut-off point (min/day)**
	15–17	.572	.442–.703	47.6	39.7	20.7
	18–30	.473	.416–.530	90.5	90.9	52.1
	31–50	.550	.502–.597	49.3	40.2	23.3
	51–65	.540	.472–.609	49.4	49.5	21.3
	**Age (years)**	**AUC**	**CI 95%**	**Sensibility (%)**	**Specificity (%)**	**Daily steps cut-off point**
	15–17	.523	.366–.680	61.9	55.9	9417
	18–30	.521	.393–.509	90.5	95.1	17,210
	31–50	.532	.485–.579	60.2	55.1	10,510
	51–65	.546	.475–.618	60.2	49.5	10,465
**Men**	**Age (years)**	**AUC**	**CI 95%**	**Sensibility (%)**	**Specificity (%)**	**MPA cut-off point (min/day**
	15–17	.390	.304–.476	1.0	61.0	50.2
	18–30	.512	.353–.672	66.7	66.1	51.3
	31–50	.572	.499–.645	70.8	56.3	38.9
	51–65	.586	.505–.668	67.7	55.1	36.5
	**Age (years)**	**AUC**	**CI 95%**	**Sensibility (%)**	**Specificity (%)**	**VPA cut-off point (min/day)**
	15–17	.890	.755–.1.0	1.0	80.5	2.2
	18–30	.600	.456–.744	83.3	51.0	0.3
	31–50	.522	.448–.595	81.5	75.6	0.5
	51–65	.476	.392–.560	82.3	79.0	0.1
	**Age (years)**	**AUC**	**CI 95%**	**Sensibility (%)**	**Specificity (%)**	**MVPA cut-off point (min/day)**
	15–17	.415	.328–.502	1.0	58.5	50.5
	18–30	.525	.365–.685	66.7	64.3	51.3
	31–50	.573	.500–.647	70.8	55.2	38.9
	51–65	.586	.505–.668	66.1	55.1	36.5
	**Age (years)**	**AUC**	**CI 95%**	**Sensibility (%)**	**Specificity (%)**	**Daily steps cut-off point**
	15–17	.496	.408–.586	1.0	50.4	9960
	18–30	.525	.374–.677	83.3	65.2	13,221
	31–50	.559	.485–.634	58.5	51.1	10,598
	51–65	.608	.531–.686	59.7	49.7	10,445

AUC, area under the curve; CI 95%, confidence interval; MPA, moderate physical activity; VPA, vigorous physical activity; MVPA, moderate-to-vigorous physical activity.

The cut-off points established for MVPA (min/day) according to BMI presented lower values than the WHtR and WHR in men and women. In the analysis by gender, men demonstrated higher cut-off points for daily steps than women in all age categories according to BMI, WHtR, and WHR. In the case of MVPA, the cut-off points for the male group were only higher than for women by WHtR and WHR, but not by BMI. Regarding age groups, the lowest cut-off point for MVPA was observed in the age group of 31–50 years (15.1 min/day) in women and in the adolescent group in men (15.4 min/day). In the case of daily steps, the adolescent group (15–17 years) showed the lowest cut-off point according to BMI, WHtR, and WHR in both sexes, both in men (5162) and in women (6143) (Tables 3, 4 and 5).

## Discussion

This study aimed to establish cut-off points for PA that identify overweight/obesity by sex and age groups in people from eight Latin American countries. The main results showed significantly higher BMI values in women than in men in all age groups, just as previous studies have shown a higher prevalence of obesity, high waist circumference, and the development of chronic diseases in non-Hispanic women^33, 34^. In general, men performed higher average PA (MPA, VPA, MVPA and number of steps) than women in all age groups. Likewise, other studies have concluded that a lower average daily PA characterizes women compared to men, especially at older ages^35^, proving to be less physically active, with lower average levels of light, moderate, and vigorous PA, and total PA^19^. The above may be attributable to a cultural issue that stablish a strong Latin American role of women in household and family-related activities, leading to less leisure time, lower engagement in PA and higher sedentary behavior in women compared to men, which can lead to a higher development of obesity-related diseases at early ages. The foregoing coincides with the analysis of this study, where the lowest average daily MVPA was observed in older women (51–65 years) according to BMI, who are characterized by carrying out light, mainly domestic activities, over more intense PA. In contrast, men tend to engage in vigorous work-related or recreational activities that may protect effect against abdominal obesity^36^.

The cut-off points for MPA, VPA, MVPA and daily steps were established based on BMI, WHtR, and WHR. Evidence shows different responses to the type of exercise found between the sexes in obese adults^37^. The present study showed that women obtained lower cut-off points for daily steps than men, obtained according to BMI, WHtR, and WHR, as well as for MVPA, according to WHtR and WHR, suggesting that men will need to comply with a greater amount of minutes of MVPA and daily steps to achieve a protective effect against overweight/obesity. Likewise, although participation in 150 min or more of MPA has been associated with a reduction in the odds of abdominal obesity, only in women it is associated with lower odds of being overweight/obese^36^.

Previous studies have established the relationship between the number of daily steps and a lower risk of all-cause mortality^16, 38^. Although seems that more steps per day is associated with lower mortality risk, specific cut off points around 8000 steps per day were identified to screen overweight^39^. Thus, our findings expand previous evidence for different sexes and age groups, suggesting a cut-off point according to BMI between 9544 and 11,412 daily steps for women and 10,295–12,509 daily steps for men in adults (31–50 years) and older adults (51–65 years) respectively, which are within the recommendations for the prevention of overweight/obesity and other related health problems. On the other hand, this study showed the lowest cut-off point for daily steps according to BMI, WHtR, and WHR in the adolescent group (15–17 years), both in men (5162) and women (6143), unlike the number of 14,414 and 11,355 suggested by a Brazilian study based on waist circumference measurements^40^. This group could have a protective factor associated with age based on the fact that overweight/obesity could be related to cellular processes similar to aging, which is associated with an increase in the percentage of body fat of approximately 1% per decade^41^.

Some limitations need to be considered, such as the study's cross-sectional design. Because the study was performed only among urban people, the results cannot be generalized to rural inhabitants. Also, accelerometers do not adequately capture some cycling and static exercise activities. These rule out certain types of movement that are not perceived and mainly measure accelerations translated into an activity count but do not discriminate in the type and/or intensity of PA performed. However, the study's strengths include that it was carried out considering the extensive database with participants from eight different countries in Latin America. In addition, in this study, the analysis was carried out by sex and age range, which allows for a more limited perspective of the needs of each age group in relation to PA and prevention of overweight/obesity. Moreover, these results presented by sex and age categories, represent a novelty for the prescription of specific PA for each group, contributing to reduce the high rates of obesity in Latin America and thus avoid the prevalence of chronic diseases associated with overweight from early ages and throughout the life cycle, improving people's functional capacity and quality of life. Lastly, better prevention of obesity will contribute to improvements in health programs implementation and the use of health resources of each nation for more preventive than curative purposes.

## Conclusion

The present study showed significant differences in all age categories between the sexes for WHtR, WHR, MPA, and MVPA. Women presented lower cut-off points for MVPA and daily steps than men to prevent overweight/obesity. In addition, the lowest cut-off point for daily steps was established in the adolescent group for both sexes and the highest in the older age groups in women, and adults in men. This research suggests that a universal recommendation for PA and daily steps is not enough and should be adjusted by sex, age range, and geographic region. Further research is required to establish the association between compliance with PA recommendations and the prevalence of other diseases associated with overweight/obesity.

## Data Availability

The datasets created and used for the current study are available from the corresponding author upon reasonable request. However, these are not publicly available due the terms of consent/assent to which the participants agreed. Please contact the corresponding author for further data and materials details.
